# Clinical photographic observation of plantar corns and callus associated with a nominal scale classification and inter- observer reliability study in a student population

**DOI:** 10.1186/s13047-017-0225-2

**Published:** 2017-10-12

**Authors:** David R. Tollafield

**Affiliations:** Spire Hospital Little Aston, Little Aston Hall Lane, B7 3UP, Sutton Coldfield, West Midlands UK

**Keywords:** Corns, Callus, Human papilloma virus, Debridement, Descriptor, Observer rater, Kappa statistic, Expert panel

## Abstract

**Background:**

The management of plantar corns and callus has a low cost-benefit with reduced prioritisation in healthcare. The distinction between types of keratin lesions that forms corns and callus has attracted limited interest. Observation is imperative to improving diagnostic predictions and a number of studies point to some confusion as to how best to achieve this. The use of photographic observation has been proposed to improve our understanding of intractable keratin lesions.

**Methods:**

Students from a podiatry school reviewed photographs where plantar keratin lesions were divided into four nominal groups; light callus (Grade 1), heavy defined callus (Grade 2), concentric keratin plugs (Grade 3) and callus with deeper density changes under the forefoot (Grade 4). A group of ‘experts’ assigned from qualified podiatrists validated the observer rated responses by the students.

**Results:**

Cohen’s weighted statistic (k) was used to measure inter-observer reliability. First year students (unskilled) performed less well when viewing photographs (*k* = 0.33) compared to third year students (semi-skilled, *k* = 0.62). The experts performed better than students (*k* = 0.88) providing consistency with wound care models in other studies.

**Conclusions:**

Improved clinical annotation of clinical features, supported by classification of keratin- based lesions, combined with patient outcome tools, could improve the scientific rationale to prioritise patient care. Problems associated with photographic assessment involves trying to differentiate similar lesions without the benefit of direct palpation. Direct observation of callus with and without debridement requires further investigation alongside the model proposed in this paper.

## Background

While photography offers a common method for assessing wounds [1], no published evaluation has been applied to plantar forefoot corns and callus. Pain associated with increasing epidermal skin thickness and concentrated areas of keratin have been associated with corns, callus and infection of the skin by human papilloma virus (HPV). During the mid-twentieth century keratoma, often described as intractable plantar keratoma (IPK), was popularised by foot surgeons in North America where an unofficial six-stage classification included viral warts (human papilloma virus or HPV) [2].

Confusion associated with HPV has afforded debate from clinical observation alone. When 43 cases were reviewed after circular excision, recurrence showed 51.1% of excised corns were associated with HPV [3]. Many professionals believe they can determine the difference between corns and verrucae and yet it is clear that clinical presentation is not always sufficient to secure an accurate diagnosis without biopsy.

It is acknowledged that there are neurological and vascular anomalies within callus [4], and human papilloma virus has provided one source of dermo-epidermal junction (DEJ) disturbance. The contribution of callus at deeper tissue level has been associated with the rupture of synovial sacs below the DEJ [5]. The public have complained that hard skin and corns return after treatment [6]. Sufficient evidence exists to highlight the shortfall in managing callus by debridement [7–9], although the use of orthoses has provided greater longevity using time related visual pain scale measurement [8, 10, 11]. Callus debridement analysis has predominantly been carried out on diabetic and rheumatoid groups rather than healthier populations where callus and corn management is part of core podiatry [12]. Thickened epidermal tissue as ‘callus’ and ‘painful’ has been described without a specific location and can lack adequate descriptive narrative [13, 14]. Annotation (of skin changes) within clinical records should include colour, border variation, symmetry within lesions, and localisation of corns/callus based on standard dermatological texts. Patterns seen in elderly patients were best represented to include lesions outside the metatarsal head (MTH) perimeter [15] but this was far from the case in similar papers.

A graded classification system came about as part of a study involving 1700 patients. The classification model allocated whole numbers without sub-divisions, with the scale graded 1–4 for plantar callus/corn presentation after hallux valgus surgery; [16], Table 1.

**Table 1:** Students reviewed 1700 patients (2000 feet) during a study in 1984 at Birmingham School of Chiropody (now relocated) associated with hallux valgus and plantar callus [16]

Grade 1	Grade 2	Grade 3	Grade 4	Grouping
59	35	6	6	Females (all)
40	29	8	12	Males (all
53	33	6	7	All combined
7	0	7	0	<10 years
24	10	2.5	1.6	<20 years

The simplified descriptor (Table 3) established the criteria for grading [16].

Fewer grade 3 and 4 lesions were found compared to grade 1 and 2 [16]. Although children were included, such lesions identified were more likely due to HPV infection. The original data capture isolated those under 10 and those collectively under 20 years (Table 2). An assumption was made that grade 4 lesions were worse than grade 1,2 and 3. It was reasoned that callus could be divided into four clear entities as distinct from viral warts, but clinical histological evidence has suggested HPV infection arising at the basal layer cannot be excluded where the constituent epidermal layers and dermal papillae are altered [3]. Further review of histology and plantar keratin is outside the remit of this paper.

**Table 2:** Assigned scores for photographic lesions were validated by experts A-E = podiatrists, F = scientist, and summated in the dominant column

Order of Lesions	A	B	C	D	E	F	Dominant	Originally assigned (DT)
1	***3***	***3***	***3***	***3***	***3***	***3***	3	3
2	***1***	***1***	***1***	***1***	***1***	***1***	1	1
3	***4***	***4***	***4***	***4***	***4***	***4***	4	4
4	1	***2***	***2***	***2***	1	***2***	2	2
5	2	***4***	***4***	***4***	***4***	***4***	4	4
6	***4***	***4***	***4***	3	***4***	3	4	4

Figures in bold and italics show greatest agreement. The original assigned score is shown in the right hand column

Podiatric classification systems have been cited without reliability studies [18–20] and extended their grading to include grades 5 and 6, where the latter related to epidermal breakdown. Since the original model for grading callus only a single paper has applied the method to clinical research and observation [21]. In regard to the descriptor used for grades 1 and grade 2 callus, the author felt lack of clarity regarding density changes between different callus. This paper appears as the first academic review of the graded system but considered other forms of physiological related pathology [22]. However, it is unclear from photographic plates provided in two papers [21, 22] that by seeking further clarification in respect to thickness, grade 2 lesions may have been confused with grade 4 lesions because the original descriptor source used was too brief [23]. Classification should be precise and reproducible. When cataloguing any keratin lesions pathogenic changes should be mentioned within the narrative. Reliability has more to do with an assessment method free from measurement error [24]. The clinician requires cost-effective and reliable systems that do not detract from clinical output.

A 32-year review of the original approach to classification of corns and callus has been considered for further evaluation in a controlled study. While this study has not been critically reviewed, one paper did consider the effect of hallux valgus on changes in the skin with callus under the forefoot. Forty-nine percent of patients the original study of 1700 patients group showed callus under the second MTH [16] while in a similar study for hallux valgus published 14 years later 34% presented with callus for the same location in a group of 104 patients [17]. While no classification was provided in this Korean Orthopaedic paper the interest shown in a similar study was helpful. Due to perceived limitations hypothesised with brief descriptors a reworked descriptor was introduced into the method.

## Method

### Pilot study & expert panel selection

Two pilot photographic studies were carried out 2013–2014 at two national conferences by consensual agreement with the organisers and participants. The observer raters were all qualified podiatrists. The first pilot study included an introduction and descriptors while the second study relied on descriptors alone. The second pilot study invited original observer raters with scores 80% + for the same photographs to review a different set of photographs. Six observer raters scoring 83%, (5/6 photographs) were accepted as ‘experts’. Five podiatrists (*skilled)* together with one biophysics engineer were recruited into the study (*n* = 6).

**Fig. 1:**
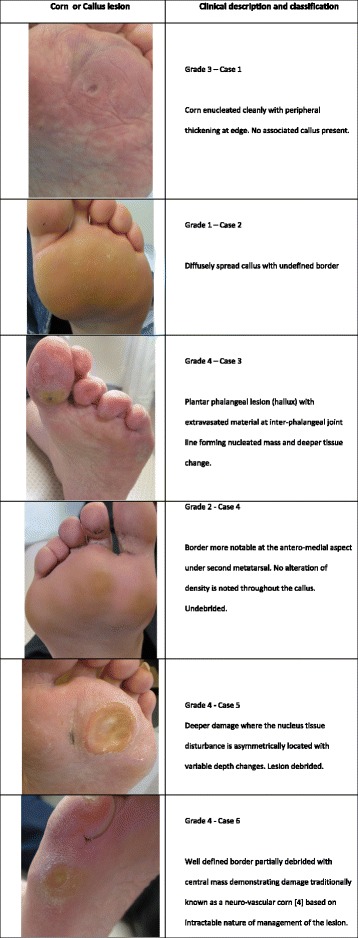
Six colour plates used in the pilot and Method by students and experts. All plates anonymised and selected by (DT) with patient consent

All students were resident at first and third year level at a Podiatry School within a University Department of Health Sciences and selected by an appointed tutor. Students were recruited along the same lines as for skilled observer raters without previous knowledge of the model grading method [25, 26]. PowerPoint™ was used to present 6 slides for student observer raters (Fig. 1) in a classroom and all anonymised sheets were returned to a podiatry tutor. First year students (*n* = 31) were inexperienced (first semester) and termed *unskilled*. Third year students (*n* = 24) had some clinical experience and but had just completed their second year and were considered *semi-skilled*. The skilled observers were used to validate the photographic lesions independent of the researcher (Table 2).

Descriptors were designed around the original paper [16] but extended to improve interpretation of the keratin presentation under the plantar surface (Table 3).

**Table 3:** Simple and detailed descriptors. The simple descriptor was utilised in the pilot studies. The detailed descriptor was designed for inexperienced students

Grade	Simplified Descriptor	Detailed descriptor
Reference only	No callus lesion. Normal	No lesion. Even colour, thickness & consistency remain within normal limits for each part of the foot. Heel, sole and pulp of toes may be thicker. There would be insufficient epidermal tissue to debride without affording damage. There are no ridges, fissures or deep tissue changes or lesions within the skin. Keratin lesions associated with other forms of hyperkeratosis do not form part of plantar callus classification.
1	No border definition but retained uniform keratin depth. Ridged or pinch callosity can be considered within the Grade 1 definition	The epidermis is thickened and may have some irregular deeper density changes so as to alter the colour. Callosity shows no border symmetry and maybe diffusely spread without any concentrated area of keratinisation. Petechiae (blood vessels) may be seen or extravasated content. Pinch callosity, also known as ridging, is callus on the edge of the forefoot, occasionally sulcus, heel or apex of a toe. The border may appear isolated as streaky (striated) callus. While this grade of callus may have a defined border it is considered Grade 1 because it conforms to physiological build up or deformity, and the deeper tissue changes are not involved as in Grade 2 or Grade 4.
2	Border definition was present or partially present with variable keratin depth. No discrete distribution of concentrated keratin is evident in the Grade lesion but asymmetric density changes might be observed	A thickness of epidermis forms usually over one or more metatarsals or phalangeal surface of a toe. The border is discrete and may be raised forming a button or disc of thickening. If a partial border is observed, then this is classified as a Grade 2 callus. Debridement may be necessary to determine any true nucleation. The underlying callus may be spongy and can only be determined by examination. Areas of flaky skin, complicated with sub epidermal hemorrhage do not constitute a nucleus of tissue and should be disregarded. If debrided the tissue is shown to have broken down, eroded or ulcerated it no longer follows the callus classification but that of a wound.
3	Concentrations of discrete keratin plugs isolated, or in groups of lesions, generally with a diameter of less than 4 mm without background callus.	Usually a discrete circumscribed area, but may be elongated. This lesion has no surrounding callus except at the extreme border where a thickened ring or rim may exist. The lesion is mostly associated with the metatarsal plantar skin where weight bearing is reduced and fat tissue is less pronounced, often with a less tightly bound epidermis. However, the lesion may not be associated with mechanical origins and can occur due to other causes including foreign body infiltration or HPV infection. If this is a suspected HPV then it no longer follows callus classification.
4	Border definition present or partially present with variable keratin depth but demonstrating discrete distributions of concentrated keratin greater than 4 mm diameter within the callus	The callus will have a circumscribed symmetrical or asymmetrical area of greater depth, ridge or greater concentration anywhere within the callus. The size can vary from lesion to lesion-occupying crater like areas after debridement. The nucleus does not have to be limited to the centre and can in some cases manifest within a larger percentage of the lesion. On debridement the base (*DEJ*) may be damaged as well as uneven in depth. As Grade 4 calluses are considered typical of intractable lesions, these are often complicated within the dermo-epidermo junction. Extravasated material, without debridement confirmation cannot be assumed consistent with Grade 4 lesions, but there may be density changes within the callus complicated by blood vessel disturbance. The same rule applies if the dermis is breached leading to a wound.

### Materials

Photos used in the PowerPointTM slides were taken using a Canon Powershot SX50HS with macro settings and standard lighting control without flash photography set at the highest definition. Appropriate patient consent was taken. Poor quality slides were removed following two pilot studies. All plates contained no facial recognition and anonymised to observer raters.

## Results

Reliability was expressed as a value of weighted quadratic kappa statistic for observer ratings on a nominal, or ordinal scale graded 1–4 [27]. A contingency table calculated the frequency of agreement and disagreement for each lesion. The strength of agreement for *k* = 0.81-1.0 implied an almost perfect state, *k* = 0.41-0.60 moderate, *k* = 0.21-0.40 fair and *k* = 0.10-0.20 slight [28]. Values of the quadratic weighted statistic obtained alongside percentage responses are reported (Table 4).

**Table 4:** Results from observer raters for students and experts (including scientist F)

	Weighted kappa	Percentage observations correct
Expert	0.88	83.0%
Year 3	0.62	56.3%
Year 1	0.33	41.9%

## Discussion

First year students demonstrated lower ability when observing photographs (*k* = 0.33). While most students observed >1 out of the 6 slides for correct observation, the majority of the student observers achieved 33-67% correct scores possible with 22% scoring 83.3% or above. The Case slide 4 proved more difficult amongst expert raters. This consisted of a lesion with a partial border under the second metatarsal head. Lack of visual depth perception could mislead the observer when considering the edge of any epidermal thickening. Partial or whole borders were intended to be interpreted as grade 2. Location would ultimately play a significant part as would the presence of an adjunctive deformity in any of the toes. Further work for post-debridement assessment is required to consider any impact on the classification model. One potential value of debridement is the ability of the skilled clinician to expose the deeper level of the epidermis to assay underlying pathology invoked by DEJ disturbance. The presence of underlying cysts and bursae however may not be exclusive to grade 1 or 2 keratin lesions [5].

Photography has been applied to a number of observation projects with musculoskeletal research using Cohen’s Kappa statistic for categorical data [28]. While other studies have used interclass correlation coefficient (ICC) statistics for reliability, Cohen tried to account for some of the errors in measuring observation reliability with percentages [29]. Reliability is related to lack of variation in a classification system when it is repeated [29, 30]. Intra-reliability observation was not studied in this project but it has been considered that inter-observer ratings reflect better reliability [31].

In one study covering wounds caused by burns, 11 observer raters presented with different skills experience. Reliability increased with experience [26]. The observer reliability of podiatry students holds true as experience increases (*R* = 0.98), taken from the k values in this study.

Student’s previous academic experience was broken down into 7 categories, but lead to no correlation in regard to ability. While the study suggested greater reliability from qualified podiatrists spread over a greater geographical area, better control was sought within an educational setting. The experts provided contrast to students’ results and were more consistent for the small panel selected. The experts achieved a reasonable outcome (*k* = 0.88/83%). Based on kappa the value of the observational system with photographic evidence alone appears reliable within the context of fitting in with descriptors (Table 3). Without the use of additional tools such as the Foot Pain and Disability Index (MFPDI) [32] clinical validation would have to be assessed further.

Wound classification observer studies have been used by expert panels to assist observation of other raters. The weighted quadratic kappa (k) statistic assists with the differentiation between poor, moderate and good observation scores. Pairs of nurses using inter-observer classification rating *k* = 0.81 – 0.97 for ulcers, faired less well when working independently *k* = 0.49 [30]. Podiatrists usually work alone but may have shared information in the classroom based exercise.

Comparable photographic reliability results were higher for experts at 0.83 in this study, and other studies using the same approach; 0.87 [25] and 0.91 [26]. Inexperienced observers in this study reached a mean 0.33 – 0.62. In contrast, nurses scored 0.33 [30], suggesting any value below 0.59 was less satisfactory for wound observation. Methodology from wound studies could not be directly compared to corns and callus [25, 26, 30] although values of k = 0.45 – 0.75 were ‘fair to good’ [26].

The hypothesis upon which four nominally graded options for corns and callus were based involved ‘staging’ to show the critical nature of lesions with and without hallux valgus deformity [16]. While no evidence of staging for epidermal thickening exists in the literature, skin that blisters following shoe rub can alter with epidermal thickening. While some resistance has been offered to expand the grades further, errors could arise if the choice of selection becomes blurred. Where seven grades for shearing callus were used for pedal skin, classification became impractical when transferring definition from text to clinic [18]. This was also found in paediatric dental study where 10 levels were used. Observer raters observing enamel damage in paediatric teeth with photography fared less well when relating to degrees of *enamel trauma* rather than *colour variation* [33]*.* Use of extensive lists of classifications, where the descriptor has large numbers of different options can weaken the method’s effectiveness. Eight stages of classification used to describe fingertip injuries produced poor observational results [24].

It is acknowledged that while more options might allow for easier classification not all lesions would be possible to classify into four categories. It would be unlikely, given both pilot study results and controlled study results, that 100% reliability could be achieved. While errors would not have significant consequences if keratin classification was mistaken, the key contribution could add to diagnostic unpredictability unless combined with reliable tools to provide a quality-related tool.

No one lesion is the same, and DEJ pathology varies widely, as the dimensions of depth change according to sub-dermal damage [26]. Inevitably this makes assigning lesion grading more difficult. In a study where photographic observation of wounds included pressure ulcers, a large proportion of photographs were not stageable, even by the experts. This was often because eschar covering the wound made it impossible to judge the extent of tissue involvement. Where extravasation arises within dense keratin overlying callus, skilled debridement ensures the DEJ has not been penetrated. It is at this point that new judgement and appropriate management is considered.

Clinical examination may reach a finite point where lesion differentiation cannot be made conclusively, whether by direct observation or from photographs without debridement. In this regard there is no contention that the use of a classification system will answer the clinician’s problems in isolation. Variations such as verrucae, fissures and pitted keratolysis must be excluded to avoid extending any unintentional inclusion with the model. However, from recent analysis of excised lesions [3], the exclusion of HPV infections will have to be reconsidered by all clinicians involved in skin management and may need to be included within the descriptor. Furthermore, once the DEJ is breached, thus forming first an erosion, then an ulcer, a different system of classification should be assigned as new pathology enters the equation.

It may be reasonable to avoid using any classification model where too many conditions become enveloped under one ‘umbrella’ system. Prognosis and outcome could be underpinned by classification provided that quantitative methods are added, e.g. visual analogue scale for pain and an assessment based on a validated health tool. Confounding errors arise more readily from photographs if descriptors used to judge lesions provide ambiguity. The difference between percentage of fibrin to cover the wound versus area of epithelisation demonstrated this aspect of observation [25, 26]. Boundary definition and callus density within the lesion appears to suffer similar errors.

Debridement as cyclical treatment has been considered an important component of ‘Core Podiatry’ [12] but fails to make a compelling argument for continuance without change based on evidence where debridement demonstrates unsustainable improvement in pain unless repeated for the low risk categories [7–11]. Paradoxically avoidance of cyclical management will offer more attraction to commissioners of health care. Inevitably classification could help to prioritise patient management of callus but without validation from other analytical methods, predictable outcomes will remain challenging.

## Conclusion

Considerations for classification have been revisited after a 30+ year period to highlight weaknesses within existing clinical healthcare models for corns and callus, especially within the NHS. Used alone, classification remains limited but may provide a method to show improvement or deterioration. When considered with good quality dermatological description and assessment quality of life, the clinician could use triage by patient questionnaire and photographic media to improve consultations. Problems associated with photographic assessment involves trying to differentiate two similar lesions using a flat or 2-D representation without the benefit of direct palpation.

Classification does not differentiate other pathology such as foreign bodies, fibrous changes within the DEJ, inclusional cysts, bursae, effects of disrupted metatarsophalangeal joints, HPV and neoplasia. A descriptor should cover all possibilities, but dermatological lesions unrelated to surface pressure or DEJ damage can obfuscate the clinician’s selection.

Reliability with observation within health must be considered important when the impact of the model used is sensitive enough to make a difference. The skill when annotating the four-point grade model depends on minimising ambiguity around border definition and recognising density changes within callus. Grades 1–4 while independent of each other could define treatment objectives by combining other tools validating impact scores and establishing underlying causes.

Kappa values for observational reliability >0.8 might provide an acceptable value for benchmarking photography, but prior tuition is important. Direct clinical observation might improve the chances of observer reliability over photographic plates.

## Abbreviations

DEJ: Dermo-epidermal junction
HPV: Human papilloma virus
ICC: Interclass correlation coefficient
IPK: Intractable plantar keratoma
MTH: Metatarsal head
